# Coaching for Emotional Resilience and Reflective Growth: Applying the University-Based Coaching Framework in Pre-Service Teacher Supervision

**DOI:** 10.3390/bs16030330

**Published:** 2026-02-27

**Authors:** Dana Morris

**Affiliations:** School of Education, The University of Texas at Tyler, Tyler, TX 75799, USA; danamorris@uttyler.edu

**Keywords:** reflective practice, teacher development, emotional resilience, pre-service teacher education, supervisor coaching

## Abstract

Teacher preparation is an emotional as well as a cognitive process in which pre-service teachers must develop both reflective judgment and the emotional resilience needed for demanding instructional contexts. This study examined how university-based supervisors enacted the relational spaces of the University-Based Coaching Framework (UBCF) and how these enactments shaped pre-service teachers’ emotional and reflective development. Drawing on qualitative analysis of coaching discourse among three supervisor-pre-service teacher pairs, the comparative case study identifies distinct coaching identities that emerged from supervisors’ patterned relational moves. These identities corresponded to varying intensities of UBCF space enactment and produced differential pathways through a reflective-motional cycle connecting appraisal, coping, and reappraisal. Findings demonstrate that supervisors’ relational stance functions as both cognitive scaffolding and as an emotional regulator. By conceptualizing UBCF-based coaching as an interactional process that integrates relational attunement with reflective challenge, this study contributes new insight into how emotional and cognitive dimensions of supervision jointly support teacher knowledge development and early professional resilience.

## 1. Introduction

Learning to teach is a complex and deeply human endeavor that requires navigating uncertainty and the continual reconstruction of knowledge through practice. Pre-service teachers must simultaneously acquire pedagogical content knowledge and develop the emotional capacity to manage the challenges inherent in early teaching experiences (Gess-Newsome et al., 2019; Loughran, 2019; Shulman, 1987; van Driel et al., 2014). These dual demands make emotional wellbeing a central, yet often underexamined, dimension of teacher education (Dreer, 2023; Hascher et al., 2021).

University-based supervision, particularly coaching grounded in reflection and feedback, plays a pivotal role in supporting both emotional and professional growth for pre-service teachers (Atkinson et al., 2021; Beltman et al., 2016). Effective coaching fosters psychological safety, enabling novice teachers to examine classroom situations openly and transform those experiences into actionable professional knowledge (Korthagen et al., 2016; Jennings & Greenberg, 2009). When reflective dialog is paired with emotional attunement, supervision becomes a regulatory process in which wellbeing and knowledge construction reinforce one another.

The University-Based Coaching Framework (UBCF) (Morris & Ogodo, 2024) provides a conceptual structure for analyzing this process. It delineates three interconnected relational spaces, identified as the secure space, the collaborative space, and the developmental space, that describe how coaching evolves from establishing trust, to co-constructing meaning, to fostering autonomy. Each space represents a developmental shift in both reflective sophistication and emotional regulation. Within these spaces, feedback is intentionally aligned with the teacher’s cognitive and affective readiness, creating conditions for sustained growth.

To deepen understanding of how reflection and regulation intersect, this study operationalizes the Aligning Wellbeing and Resilience in Education (AWaRE) Model (Hascher et al., 2021), which conceptualizes resilience as a cyclical process involving emotional appraisal, coping and strategy activation, and reappraisal and wellbeing restoration within a distinct contextual environment. When mapped onto the UBCF, these practices mirror how teachers experience and process coaching interactions.

By merging frameworks, the study reframes teacher knowledge development as both a cognitive and emotional process where knowledge is not transmitted from coach to teacher but constructed through cycles of reflection, feedback, and emotional regulation (Atkinson et al., 2021). By analyzing coaching conversations through this integrated lens, the paper contributes to the literature on teacher resilience and wellbeing as well as professional knowledge growth, offering a model for how emotionally intelligent supervision fosters sustainable professional learning in pre-service teacher education.

While the UBCF has been used to describe relational conditions within professional spaces that support pre-service teacher learning, this study extends the framework by positioning emotional regulation as a central coaching mechanism rather than a contextual backdrop. Drawing on the AWaRE model’s conceptualization of resilience as a recurring process of emotional appraisal, coping, and reappraisal (Hascher et al., 2021), the study specifies how these processes are enacted through supervisory discourse. The reflective–emotional cycle identified in this study makes visible the moment-to-moment coaching moves through which supervisors stabilize emotion, scaffold reflection, and progressively shift responsibility to pre-service teachers. In this way, the UBCF is extended from a framework describing relational space to one that clarifies how emotional regulation and reflective growth are jointly produced through adaptive supervisor coaching discourse.

By examining supervision through this lens, the study addresses coaching identity as it emerges in practice, understood not as a fixed role or personal trait, but as an enacted stance shaped by relational context, teacher readiness, and “in-the-moment” sensemaking within coaching conversations.

Although research on teacher reflection, instructional coaching, and teacher wellbeing has grown substantially, these bodies of literature have largely developed in parallel, with little regard to how emotional regulation and reflective growth are jointly produced through coaching discourse within supervision. By integrating the AWaRE model with the UBCF, this study posits emotional regulation not as a contextual condition or individual capacity, but as a mechanism through which reflective depth and professional learning are scaffolded in supervision. Thus, the study advances conversations in teacher education and coaching by offering a process-oriented account of how coaching practices simultaneously support reflective knowledge development and emotional resilience in pre-service teachers.

Guided by this integrated perspective, the purpose of this study was to examine how university-based supervisors enacted the spaces of the UBCF and how these enactments shaped pre-service teachers’ emotional and reflective knowledge development. Using the combined lenses of the UBCF, the AWaRE Model, and the resulting reflective–emotional cycle conceptualized in this study, the researcher sought to identify the coaching identities that emerged across cases and to understand how distinct supervisory approaches fostered reflective growth and emotional regulation during post-observation conferences. The following research questions guided this inquiry:

1. How do supervisors’ enactments of the secure, collaborative, and developmental spaces of the UBCF give rise to distinct coaching identities in university-based supervision?
2. How do these coaching identities shape pre-service teachers’ emotional regulation and reflective development across cases?

## 2. Theoretical Foundations and Review of Literature

This study is grounded in an integrated theoretical foundation that brings together Reflective Practice (Schön, 1983) and the conceptual frames of the AWaRE Model (Hascher et al., 2021) and the UBCF (Morris & Ogodo, 2024). Each framework contributes a distinct but complementary perspective for understanding how pre-service teachers learn and adapt emotionally and professionally within university-based coaching relationships. Together, they conceptualize learning as a relational and affective process where reflection and emotional regulation operate as mutually reinforcing mechanisms of teacher growth.

Existing research on teacher reflection tends to promote cognitive processes, often neglecting the emotional labor and regulation required to sustain reflective engagement (Smirnova, 2024; Boud & Walker, 1998). At the same time, research on teacher wellbeing has largely focused on stress reduction or coping strategies, overlooking the relational mechanisms through which wellbeing supports learning (Day & Gu, 2014; Schlosser & Paetsch, 2023; Zhang, 2023). Furthermore, few studies have explored how these dimensions operate together as a dynamic system through which reflection, feedback, and emotional regulation work together to advance professional knowledge and resilience among teachers. This study aims to fill the gap by situating reflection as a dynamic professional learning process in which emotional regulation, relational feedback, and supervisory interactions work together to support teachers while developing knowledge and resilience.

### 2.1. Reflection as a Core of Professional Learning

Reflection has long been recognized as both the intellectual and affective engine of teacher learning. Dewey (1910) first described reflective thought as a disciplined process of inquiry through which experience is reconstructed into knowledge. Schön (1983) expanded this foundation by articulating two interdependent forms. Reflection-in-action occurs in the immediacy of teaching, and reflection-on-action follows experience and enables meaning-making. Both forms require teachers to engage cognitively and emotionally with uncertainty.

Schön’s (1983) conception of reflection-in-action and reflection-on-action positions teaching as a form of practical inquiry, an act of thinking through doing. Teachers continually interpret, adjust, and respond to the unpredictable dynamics of the classroom. Reflection, therefore, represents both cognitive agility and emotional composure (Korthagen, 2017; Loughran, 2019). Pre-service teachers may feel particularly vulnerable when reflecting on teaching experiences with a supervisor. Yet, when feedback is experienced as psychologically safe, reflection can move beyond surface-level evaluation to become transformative sense-making.

Recent scholarship highlights that authentic reflection demands emotional honesty, self-awareness, and tolerance for the unknown and unexpected (Smirnova, 2024; Korthagen et al., 2016). Korthagen (2017) distinguishes between action-oriented reflection, focused on what to do next time, and meaning-oriented reflection, focused on why events unfolded as they did, how the teacher felt, and what deeper beliefs or motivations were activated. He emphasizes that reflection is not merely retrospection but emotionally mediated sense-making, in which teachers reconcile dissonance between beliefs and classroom realities. His contention is that reflection is essential for transforming experience into learning. When pre-service teachers encounter moments of failure or discomfort, emotional regulation becomes a prerequisite for learning rather than a distraction from it. Ultimately, reflection functions as the bridge connecting experience to emotion and knowledge development (Korthagen et al., 2016; Korthagen, 2017). When enacted within emotionally attuned supervision, reflection fosters the adaptive expertise necessary for teachers to navigate the complexities of practice with confidence.

### 2.2. The AWaRE Model of Teacher Wellbeing

The Aligning Wellbeing and Resilience in Education (AWaRE) Model (Hascher et al., 2021) conceptualizes teacher resilience and wellbeing as dynamically interconnected processes rather than static traits or outcomes. Within this model, resilience is positioned as a situated, cyclical process through which teachers appraise emotional experiences, select and activate coping strategies, and engage in reappraisal that can restore or enhance wellbeing. Wellbeing, in turn, is understood as both a contextual condition and an outcome of this resilience process.

Drawing on Hascher et al. (2021), this study conceptualized teacher resilience as a dynamic, situational process through which teachers draw on personal and contextual resources to adapt to professional challenges. Rather than a stable trait, resilience is understood as a capacity that is activated in response to perceived adversity and enacted through processes such as emotional regulation, reflection, and strategic action. In contrast, teacher wellbeing refers to a teacher’s subjective evaluation of their professional life, characterized by a predominance of positive over negative experiences (Hascher et al., 2021; Mansfield et al., 2016). Teacher wellbeing encompasses dimensions such as job satisfaction, emotional experience, professional fulfillment, and a sense of purpose or meaning in one’s work.

**Figure 1:**
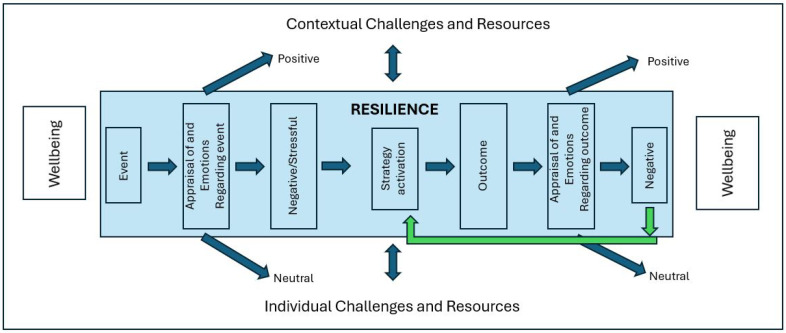
The AWaRE Model for Teacher Wellbeing adapted from (Hascher et al., 2021).

The relationship between resilience and wellbeing in the AWaRE model is therefore one of process and reciprocal influence. Resilience functions to maintain, restore, or develop wellbeing in the face of professional challenge, while wellbeing shapes teachers’ capacity to appraise situations constructively and activate adaptive coping strategies (Hascher et al., 2021). Teachers with stronger baseline wellbeing are better positioned to interpret stressors as manageable and to persist through difficulty. Conversely, successfully navigating challenges through resilient action reinforces wellbeing through meaning-making, mastery, and affirmation (Zhang, 2023). Figure 1 illustrates this cyclical relationship between resilience and wellbeing as represented in the AWaRE Model.

### 2.3. The University-Based Coaching Framework Model

The UBCF (Morris & Ogodo, 2024) integrates key features of cognitive coaching (Costa & Garmston, 2015) and real-time coaching (Stahl et al., 2016), including iterative feedback cycles, reflective dialog, and adaptive, learner-centered support. However, UBCF extends these approaches by placing a more explicit emphasis on emotional attunement and responsiveness to pre-service teachers’ developmental and affective needs. Within the UBCF, coaching unfolds across three interconnected relational spaces:

- Secure Space—where psychological safety enables vulnerability and emotional appraisal;
- Collaborative Space—where shared inquiry and feedback dialog support coping and co-regulation;
- Developmental Space—where guided autonomy and challenge promote reappraisal and growth.

**Figure 2:**
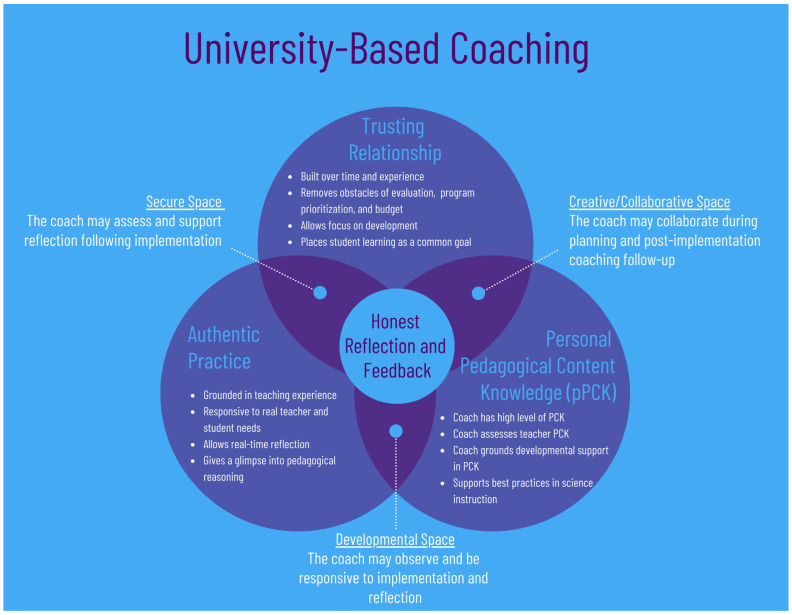
The University-Base Coaching Framework reformatted from (Morris & Ogodo, 2024).

As illustrated in Figure 2, the framework’s structured yet flexible design provides an effective foundation for fostering reflective, adaptive, and resilient educators.

Together, the secure, developmental, and collaborative spaces create an ecosystem that nurtures reflective thinking, emotional regulation, and professional growth for pre-service teachers. As an emotionally attuned approach to professional growth, the UBCF positions coaching as both instructional and relational balancing cognitive challenge with emotional support. This dual emphasis enables pre-service teachers to engage deeply in reflection, transform experience into learning, and cultivate the resilience needed to sustain practice across their careers. Ultimately, the framework illustrates that reflective growth and teacher wellbeing are interdependent outcomes of effective university-based coaching.

In the spaces of the UBCF, reflection is enacted as a dialogic process between supervisor and pre-service teacher. Coaches model empathy and curiosity, framing feedback as inquiry rather than judgment (Morris & Ogodo, 2024). This form of relational reflection is deeply tied to emotional attunement and trust, conditions that allow pre-service teachers to explore conflict and uncertainty productively (Costa & Garmston, 2015; Knight, 2015, 2018). Reflection thus functions as both a cognitive bridge linking experience to theory and an emotional bridge linking vulnerability to resilience. Through emotionally attuned dialog, pre-service teachers learn to interpret feedback, regulate emotions, and reconstruct professional knowledge in emotionally supported contexts.

### 2.4. Adaptive Feedback and the Developmental Process of Learning to Teach

Feedback is not only informational but also a form of emotional communication that signals safety, respect, and professional regard. Research consistently demonstrates that feedback is most effective when it is actionable, grounded in observed practice, and delivered within a trusting relationship (Atkinson et al., 2021; Desimone & Pak, 2017). A substantial body of research further indicates that the tone, framing, and relational stance of feedback shape how pre-service teachers interpret their experiences and whether feedback leads to meaningful instructional change (Hammond & Moore, 2018; Haneda et al., 2019).

Instructional coaching employs a range of feedback approaches, including supportive, directive, and reflective forms, each serving distinct purposes depending on teachers’ developmental needs and instructional contexts. Importantly, the effectiveness of these approaches is mediated by the coach’s stance and the quality of the coach-teacher relationship, which influence teachers’ sense of psychological safety, agency, and willingness to engage in reflection (Costa & Garmston, 2015; Desimone & Pak, 2017; Haneda et al., 2019).

Within this relational context, emotional regulation functions as a cross-cutting mechanism that supports teachers’ capacity to receive and act on feedback. Coaching interactions that acknowledge emotional responses and reframe challenges help reduce defensiveness and support reflective engagement, thereby strengthening self-efficacy and professional learning (Atkinson et al., 2021; Brookhart, 2017; Korthagen et al., 2016). As teachers develop greater emotional resilience, they assume increased responsibility for analyzing evidence, setting goals, and directing their own growth (Korthagen, 2017). Thus, feedback operates as a bridge between reflection and emotional regulation, serving not only as a pedagogical tool but as a relational practice central to learning to teach.

Table 1 summarizes the primary feedback approaches described in the instructional coaching literature and highlights their distinct purposes and relational functions. Importantly, emotional regulation is not presented as a discrete feedback type, but as a cross-cutting mechanism that shapes how feedback is received and enacted across approaches.

From a developmental standpoint, feedback which is adapted to the needs of the teacher supports progressive autonomy. As teachers gain emotional resilience, they assume greater responsibility for analyzing evidence and setting goals (Korthagen, 2017; Korthagen et al., 2016). Thus, feedback represents the link between reflection and emotional regulation. It becomes a vehicle for emotional growth, teaching novice and early-career educators how to process critique, reframe setbacks, and persist through obstacles. In this sense, feedback is not simply a pedagogical tool but a relational act central to learning to teach, and the most effective coaching should blend the types of feedback, aligning them with the teacher’s developmental readiness.

### 2.5. Integrating Frameworks with the Coaching Model

This integration is theoretically necessary because existing frameworks address reflection, coaching, and teacher wellbeing as related but largely separate constructs. This separation limits the explanation of how emotional regulation functions within reflective learning. The AWaRE model conceptualizes resilience and wellbeing as recurring emotional processes, but it does not specify how these processes are enacted or supported through pedagogical interaction. Coaching frameworks, including the UBCF, have primarily described relational contexts and feedback without fully imagining the emotional mechanisms through which reflective growth is produced. By integrating AWaRE with the UBCF, this study moves beyond conceptual alignment to propose a process model that explains how supervisors’ coaching moves mediate appraisal, coping, and reappraisal within reflective dialog. As a result, emotional regulation and knowledge development become co-constructed outcomes of supervision.

The UBCF integrates these theoretical and conceptual perspectives into a holistic model of reflective–emotional learning. It synthesizes Schön’s (1983) reflective adaptability and Hascher et al.’s (2021) AWaRE Model into three relationally mediated spaces through which coaches calibrate emotional and cognitive support. Within the spaces of the UBCF, emotional safety allows for vulnerability and appraisal; co-regulation fosters reflective coping; and autonomy enables reappraisal and growth. Table 2 provides an overview of the AWaRE Model components within the coaching spaces of the UBCF and the resulting functions in the reflective–emotional cycle.

**Figure 3:**
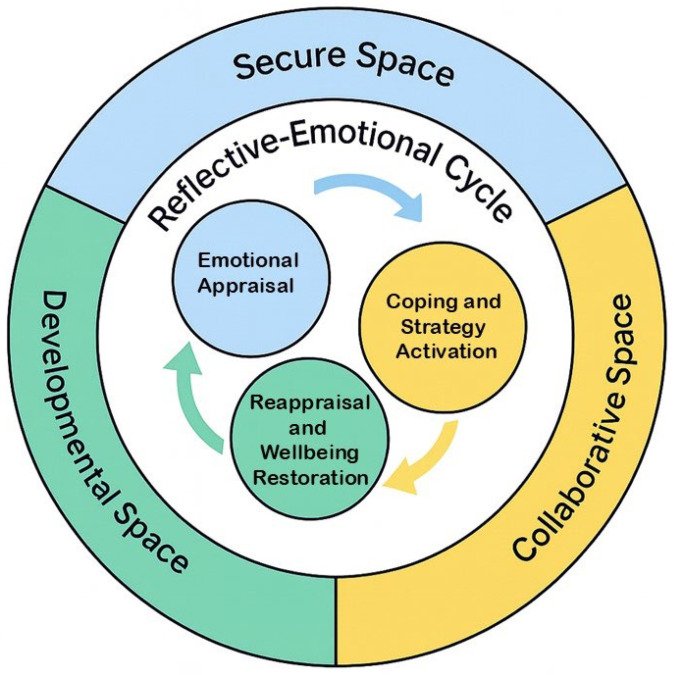
The Integrated Framework (with embedded reflective–emotional cycle). Note: Derived from the integration of the AWaRE Model (Hascher et al., 2021) and the UBCF (Morris & Ogodo, 2024).

Figure 3 illustrates how emotional appraisal, coping, and reappraisal processes from the AWaRE model operate within the secure, collaborative, and developmental spaces of the UBCF, forming a reflective–emotional cycle that supports both teacher wellbeing and knowledge development.

By positioning emotional regulation as an integral component of reflective practice, the UBCF reframes supervision as both a cognitive and an affective process. This integrated lens illustrates how emotionally attuned coaching sustains teachers’ capacity to adapt instruction and thrive within the complex emotional environment of teaching. The current study examines how university-based coaches enact these intertwined processes of reflection, feedback, and emotional regulation. In this way, it seeks to illuminate how emotionally attuned coaching practices contribute to the development of reflective and resilient educators prepared for the emotional realities of teaching.

### 2.6. Coaching Identity as an Enacted Practice

In this study, coaching identity is conceptualized as an enacted stance that emerges through supervisors’ “in-the-moment” participation in coaching conversations. Rather than representing a static personality or coaching style, coaching identity is understood as emergent, relational, and context dependent shaped through interaction with pre-service teachers. Supervisors’ stances are influenced by teachers’ emotional cues, developmental readiness, and the immediate demands of instructional situations, resulting in adaptive shifts in how support and challenge are enacted across and within conversations.

This conceptualization aligns with Korthagen’s view of professional identity as dynamically constructed through reflective engagement with practice. Korthagen (2017) emphasizes that professional identity is not a static attribute but is continually shaped through meaning-oriented reflection on experience, particularly as educators interpret emotional, motivational, and contextual dimensions of practice and respond to situational demands. From this perspective, identity is enacted in how practitioners interpret situations and choose actions that align with their developing professional commitments. Applied to supervision, coaching identity can therefore be understood as a situated manifestation of supervisors’ professional knowing, enacted differently as teachers’ emotional and cognitive needs evolve.

Similarly, Loughran’s work on professional practice underscores that identity is formed and revealed through action over time, particularly in how educators model thinking and make pedagogical decisions visible to others (Loughran, 2019). Loughran argues that professional identity is expressed through what practitioners address, how they frame problems, and how they engage learners in reflective dialog. In the context of university-based supervision, coaching identity is enacted through supervisors’ discursive moves. In other words, who they are as a coach is seen in how they frame feedback, pose questions, and position pre-service teachers as learners. Together, these perspectives support viewing coaching identity not as a fixed trait of the supervisor, but as a situated stance that emerges through relational engagement in coaching conversations.

## 3. Methods

To guide the design and analysis of this qualitative study, two guiding statements were developed to align with the theoretical and conceptual focus of the investigation. First, the analysis explored how supervisors’ enactment of the secure, collaborative, and developmental spaces of the UBCF reflected distinct coaching identities that shaped pre-service teachers’ emotional and reflective development. Second, the study examined how supervisors’ enactments of these spaces compared across cases in fostering pre-service teachers’ emotional and reflective development during post-observation coaching. These guiding statements provided the analytic lens through which supervisory discourse was examined, positioning the UBCF as both a relational structure and a comparative interpretive framework for understanding coaching interactions.

### 3.1. Research Design

This study employed a comparative case study design (Yin, 2018) to explore how university-based supervisors enacted the UBCF spaces in ways that encouraged pre-service teachers’ emotional and reflective development and revealed distinct coaching identities across cases. A qualitative approach was chosen to capture the relational and cognitive dimensions of coaching that are central to the reflective–emotional processes embedded within post-observation conferences.

Drawing on principles of interpretivist inquiry (Schwartz-Shea & Yanow, 2012; Merriam & Tisdell, 2016), the study sought to understand how meaning was constructed through supervisors’ language and coaching stance as expressed in authentic post-conference dialogs, and how these interactions were interpreted as supporting pre-service teachers’ emotional regulation and reflective development. The comparative case design enabled analysis of how the UBCF spaces were enacted across supervisors and cases, revealing distinct coaching identities and illustrating how the framework was taken up in relation to teachers’ varying levels of cognitive and affective readiness.

### 3.2. Participants and Context

Three pre-service teacher-supervisor pairs were purposefully selected (Merriam & Tisdell, 2016) to represent distinct stages of reflective and emotional growth. All three university supervisors coached pre-service teachers enrolled in a semester-long clinical placement, during which they conducted multiple observations and conferences. Prior to the semester, all supervisors participated in a coaching professional development workshop where they were explicitly trained in the principles and application of the UBCF to ensure consistency and fidelity in its implementation across cases.

Each case was bounded by a semester-long coaching relationship between a pre-service teacher and a university-based supervisor; however, for this study, one video-recorded post-teaching conference per pair was selected as the focal unit of analysis. This session represented a concentrated moment of reflective–emotional regulation in which the supervisor and teacher jointly processed a recent lesson, negotiated feedback, and engaged in meaning-making around instructional and emotional challenges. By examining these single coaching conversations as sites of reflective–emotional interaction, the study investigated how coaching feedback intersected with trust, wellbeing, and reflective growth within the relational spaces of the UBCF.

Pseudonyms of Sylvia, Beth, and Ava were used throughout for the supervisors. Sylvia’s pre-service teacher, considered to be at novice level, demonstrated limited confidence, minimal reflective depth, and high emotional sensitivity. Beth’s pre-service teacher, thought to be at the intermediate level, displayed growing competence and emerging metacognition. Ava’s pre-service teacher presented at an advanced level and exhibited strong autonomy, reflective reasoning, and low emotional dependence. This sampling allowed cross-case comparison of how emotional attunement and feedback styles functioned at different developmental levels. All participants provided informed consent for participation and recording, with institutional approval secured.

### 3.3. Data Collection

Data were derived from three video-recorded post-conference coaching sessions, one per supervisory pair, conducted immediately following a classroom lesson taught by the pre-service teacher. Each session lasted approximately 20 to 30 min and was transcribed verbatim. Video data were chosen for their ability to capture both verbal and nonverbal expressions, including tone, pacing, and body language, that signal emotional attunement and relational quality. Following each recording, field notes and analytic memos documented contextual details such as classroom events referenced in the discussion, observable affective cues, and the supervisor’s coaching stance. Immediately after each post-teaching coaching session, the researcher met individually with each supervisor to clarify interpretations of verbal and nonverbal markers documented in the field notes. By focusing on one authentic post-lesson reflection per pair, the study isolated the moments of emotional and cognitive reconstruction when teachers and supervisors jointly interpreted teaching experiences, navigated emotional responses, and transformed feedback into professional insight.

### 3.4. Data Analysis

Data analysis followed an iterative, interpretive process of coding, theme development, and cross-case synthesis (Braun & Clarke, 2006). Transcripts were first coded to identify patterns in supervisors’ coaching moves, including expressions of: (1) supportive feedback such as validation, empathy, and reassurance, (2) directive feedback such as specific suggestions and corrective modeling, and (3) reflective engagement such as inquiry-oriented questioning and metacognitive prompts. Additional codes captured indicators of emotional regulation, including moments of reframing, acknowledgment of stress, and coping-oriented language. These codes were then mapped onto the UBCF’s three relational spaces and examined through the appraisal-coping-reappraisal lens of the AWaRE Model (Hascher et al., 2021) to illuminate how reflection and emotion operated simultaneously within coaching interactions. As themes were generated, they revealed how each relational space functioned within the emerging reflective–emotional cycle. Together, these themes illustrated how supervisors’ coaching identities shaped the ways reflection and emotional regulation unfolded for pre-service teachers. 

Data were analyzed using an iterative, inductive coding process conducted by the researcher. ATLAS.ti (version 25.0.1.32924, Scientific Software Development GmbH, Berlin, Germany) was used to manage transcripts, organize codes, and facilitate systematic comparison across data sources; however, analytic decisions and theme construction remained grounded in researcher-led interpretation. Appendix A contains Table A1, a representation of the code categories, descriptions of each category, and example codes.

Cross-case comparison highlighted how supervisors adapted their feedback strategies to align with each pre-service teacher’s developmental readiness, making visible the distinct coaching identities underlying their enactment of the UBCF spaces. To support methodical comparison across cases, analytic matrices were developed to display how each supervisor enacted the UBCF spaces and how these enactments aligned with the emotional and reflective responses of their pre-service teachers. These matrices were structured tables used to organize codes, themes, and illustrative excerpts (Miles et al., 2014). Throughout the analysis, the researcher maintained notes and memos to track interpretive decisions.

Because the study employed an interpretive, discourse-focused analytic approach, the goal was not to quantify supervisory moves through strict numeric counts but to understand their relative prominence, purpose, and interactional function within each case. Thus, the evaluation of each code category was based on its intensity, meaning the relative proportion, salience, and functional weight of each coaching move within the supervisor’s overall discourse (Braun & Clarke, 2006; Miles et al., 2014). Intensity reflects how strongly a given category shaped the supervisor’s coaching identity and the pre-service teacher’s reflective–emotional experience, consistent with analytical traditions in interpretive qualitative inquiry (Merriam & Tisdell, 2016; Schwartz-Shea & Yanow, 2012) and interactionally grounded analyses of mentoring and coaching discourse (Vedder-Weiss et al., 2025). In this study, intensity ratings captured how frequently a move appeared as well as how prominently it functioned within the interactional flow, and how consequential it was for shaping emotional regulation and reflective engagement across the UBCF spaces.

To determine intensity, each supervisor’s transcript was read holistically and coded for the presence of supportive feedback, directive feedback, reflective prompts, emotional regulation indicators, and markers of the UBCF spaces. A category was rated “High” when it occurred frequently, appeared in extended turns, or played a central role in shaping the tone and progression of the conference. A category was rated “Moderate” when it appeared consistently but did not dominate the interaction. A category was rated “Low” when it appeared rarely or did not significantly contribute to or change the tone of the conference.

To capture not only the presence of coaching moves but their relational and emotional significance, an intensity-based coding approach was used. All intensity judgments were made by the researcher through iterative engagement with the data, including repeated viewing of coaching conversations, analytic memoing, and constant comparison within and across cases. Intensity was conceptualized qualitatively, reflecting the salience, emotional weight, and relational impact of a coaching move rather than just its frequency or duration. For example, a coaching move was coded as high-intensity emotional regulation when the supervisor explicitly named and reframed a pre-service teacher’s emotional response and temporarily suspended instructional critique to support emotional processing. In contrast, affective acknowledgments that were brief or embedded within instructional feedback were coded as moderate intensity. Indicators used to support intensity judgments are summarized in Table A2 of Appendix A.

To support reflexivity and analytic transparency, the researcher maintained detailed reflexive memos documenting emerging interpretations and decision points. In addition, the researcher met with each supervisor following coaching sessions to discuss their intent, perspective, and interpretation of the interaction in relation to the researcher’s analytic view. These conversations informed reflexive sensemaking but did not function as member checking or alter analytic authority, which remained with the researcher. Consistency of intensity judgments was supported through in-case comparison of similar coaching moves and cross-case comparison of how comparable moves functioned differently depending on teachers’ emotional cues and developmental readiness.

Because the researcher had prior experience as a university-based supervisor and instructional coach, ongoing reflexivity ensured awareness of potential bias and supported careful attention to the emotional and relational nuances present in coaching conversations.

### 3.5. Trustworthiness and Rigor

Credibility and dependability were strengthened through multiple strategies to ensure the quality and transparency of the research process (Lincoln & Guba, 1986). Triangulation of transcripts and field notes allowed for cross-verification of findings and enhanced interpretive depth. Member checks with participating supervisors ensured that the interpretations accurately reflected their experiences and intentions. To support the trustworthiness of the analysis, the researcher met with each supervisor following their post-teaching coaching session to clarify interpretations of verbal and nonverbal markers recorded in the field notes. Finally, thick, contextual description and attention to the relational nuances of each case strengthened the transferability of the findings. Collectively, these measures ensured a rigorous and trustworthy portrayal of how coaching discourse, emotion, and reflection intertwined within the UBCF.

## 4. Results

The findings are presented in two parts. Part I provides individual case analyses that describe how each supervisor enacted the secure, collaborative, and developmental spaces of the UBCF and how these enactments reflected distinct coaching identities. These identities shaped the emotional and reflective development of the pre-service teachers based on their varying levels of readiness. Part II offers a cross-case comparison that highlights differences in the intensity and function of supervisors’ coaching moves across the three cases. Together, these analyses illustrate how enactments of the UBCF spaces contributed to distinct trajectories in the reflective–emotional cycle.

### 4.1. Part I: Individual Case Findings

#### 4.1.1. Sylvia: A Supportive/Stabilizing Coaching Identity

Sylvia’s coaching was characterized by a strong emphasis on emotional reassurance, normalization of difficulty, and consistent relational grounding which are hallmarks of the UBCF’s secure space (Morris & Ogodo, 2024). Her supervision of a novice pre-service teacher centered on reducing anxiety and building psychological safety. For example, she frequently used validating language which served to buffer the pre-service teacher’s emotional stress and anchor the conversation in relational trust. Emotional acknowledgments and invitations for emotional disclosure occurred with high intensity and structured the rhythm of the conversation.

Reflective prompts appeared in Sylvia’s coaching but typically in short or scaffolded forms, such as “What were you noticing?” These prompts helped initiate surface-level reflection, but they did not consistently extend into deeper analysis. Sylvia offered directive suggestions when needed, most often to correct small behavioral or procedural issues, yet these appeared less prominently than supportive talk. Collectively, these patterns reveal a supportive and stabilizing coaching identity that helped the novice pre-service teacher regulate emotions and build confidence, while providing only limited opportunities for higher-level reflective reasoning.

In Sylvia’s coaching session, supportive and emotionally regulating language was both frequent and central. Her turns included statements such as, “That’s completely normal for your stage,” and “You’re improving, even if it doesn’t feel like it,” which served to normalize stress, buffer negative emotions, and maintain psychological safety. These supportive moves were woven throughout the dialog and shaped how Sylvia responded to uncertainty or difficulty expressed by the novice pre-service teacher. Sylvia also initiated discussions by asking about emotions. For example, she asked, “You seemed a bit overwhelmed. What was causing that?” reinforcing the secure space as her dominant relational stance.

Using a reflective–emotional cycle as a lens, Sylvia’s approach is predominately characteristic of the appraisal phase. She helped her pre-service teacher name emotions, surface concerns, and validate internal states before moving into cognitive or strategic reasoning. This justified high-intensity ratings for supportive feedback, emotional regulation, and secure space indicators with low-intensity ratings for collaborative and developmental space enactment.

#### 4.1.2. Beth: A Guiding/Collaborative Coaching Identity

Beth’s supervision displayed a more balanced integration of the secure, collaborative, and developmental spaces, reflecting a guiding/collaborative coaching identity. Her discourse demonstrated a patterned sequence of “affirm-probe-suggest,” through which she maintained rapport while supporting the pre-service teacher’s emerging instructional reasoning. For instance, she began by affirming competence and then gently introduced a developmental nudge. Such moves acknowledged the pre-service teacher’s strengths while providing concrete guidance for improvement.

Beth frequently used reflective prompts to extend the teacher’s reasoning, asking questions like, “Do you think that was too many?” or “What do you think helped them engage here?” These questions supported co-analysis and positioned the pre-service teacher as an active meaning-maker. Her use of directive feedback was purposeful and targeted, typically offered after affirmations or reflective questioning. For example, she suggested, “If you’re having difficulty getting students to answer, you may want to try a pair-share first.” These moves demonstrated a collaborative stance that maintained emotional support while fostering deeper reflection. Beth’s coaching identity thus helped the pre-service teacher traverse the reflective–emotional cycle moving from tentative reasoning toward greater metacognitive awareness.

Beth’s coaching demonstrated a balanced, guiding/collaborative identity in which supportive, directive, and reflective moves occurred in roughly equal measure. Her “affirm-probe-suggest” feedback sequences positioned the pre-service teacher as both capable and still developing. For example, she affirmed instructional strengths, “I like the timer. It keeps both you and the students moving forward” and then provided a gentle directive, “You need to be a little louder, okay?” She also engaged the pre-service teacher in reflective judgment when discussing modeling. These moves reveal consistent collaborative space enactment and co-produced reasoning.

In terms of the reflective–emotional cycle, Beth’s discourse reflected movement from Appraisal when identifying emotions and uncertainties into coping and strategy activation by supporting strategic adjustments and problem-solving. Her coaching identity bridges reassurance and challenge, justifying moderate-to-high intensity ratings for reflective prompting and collaborative space indicators. Together, these moves justified moderate-to-high intensity ratings for reflective prompting and collaborative space enactment, and moderate intensity for supportive and directive feedback consistent with her identity as a guiding/collaborative supervisor who bridged reassurance and challenge.

#### 4.1.3. Ava: A Developmental/Challenging Coaching Identity

Ava’s coaching predominantly enacted the UBCF’s developmental space with high intensity, reflecting a developmental/challenging coaching identity. Her feedback focused on promoting autonomy, professional agency, and reflective depth. She employed analytic questioning that pushed the pre-service teacher to evaluate instructional decisions through a more advanced lens. Reflective prompts were frequent and generative, often initiating extended reasoning sequences that required the pre-service teacher to articulate rationales, consider alternatives, or identify opportunities for refinement.

Supportive comments were present but brief, typically used to acknowledge competence before moving quickly into developmental challenges. For example, “You’re very affirming with your students,” was followed by a deeper, analytic prompt about instructional choices. Ava also raised questions about teacher agency and structural constraints like, “Do you feel like you don’t have the freedom to change this,” which invited the pre-service teacher to articulate professional decision-making processes and emotional responses. These moves fostered emotional independence and high-level reflective engagement, enabling the advanced pre-service teacher to move through the reflective–emotional cycle. Ava’s coaching identity thus cultivated self-regulation, deeper analytic reasoning, and stronger instructional independence.

Ava’s coaching demonstrated high-intensity developmental challenges and reflective prompting. Her questions such as, “Are you feeling like you’re getting everyone’s attention?” and “Is there anything else you feel like you want to change?” were frequent, analytic, and aimed at extending professional confidence. These prompts led the advanced pre-service teacher into extended turn-taking characterized by interpretive reasoning, quickly moving the dialog into the developmental space.

Viewed through the reflective–emotional cycle, Ava’s moves primarily supported coping and strategy activation, such as helping the pre-service teacher link emotions to instructional adjustments, and frequently progressed into reappraisal, guiding the teacher to reinterpret events in constructive, agentic ways. Supportive feedback was present but brief, justifying low-moderate intensity ratings for supportive moves and high-intensity ratings for reflective prompts and developmental space enactment.

### 4.2. Part II: Cross-Case Analysis

The cross-case analysis examined patterns in how the supervisors enacted the UBCF spaces and how these enactments contributed to differences in addressing pre-service teachers’ emotional and reflective development. The analysis revealed clear differences in the intensity of supportive, directive, reflective, and relational moves across cases. This interpretive approach helped identify coaching identities that were distinct yet consistent with each pre-service teacher’s developmental readiness. As summarized in Table 3, the three supervisors demonstrated varying degrees of engagement across the UBCF spaces. Additionally, each one induced emotional regulation and enacted types of feedback and reflection prompts with varying levels of intensity.

#### 4.2.1. Variation in Dominant UBCF Spaces

The supervisors exhibited distinct patterns of relational space enactment aligned with their pre-service teachers’ developmental needs. Sylvia’s talk centered almost exclusively on the secure space, providing emotional safety and reassurance. Beth displayed the most balanced pattern, consistently moving between secure and collaborative spaces while offering moderate developmental challenges. Ava enacted the developmental space most strongly, using analytic questioning and challenge-oriented talk to deepen reflective engagement. These patterns map directly onto the reflective–emotional trajectories observed in the pre-service teachers.

#### 4.2.2. Differential Pathways Through the Reflective–Emotional Cycle

Cross-case patterns demonstrate that each supervisor’s coaching identity created a different pathway through the reflective–emotional cycle. Sylvia’s pre-service teacher showed early-stage reflective responses anchored in emotional stabilization. Beth’s pre-service teacher demonstrated mid-level reflective development shaped by shared analysis and evaluative dialog. Ava’s pre-service teacher exhibited advanced reflective reasoning and emotional autonomy supported by challenge-oriented discourse. The differing pathways suggest that the UBCF spaces provide a flexible relational structure that adapts to pre-service teacher readiness levels.

#### 4.2.3. Differences in Coaching Identities Across Cases

When examined together, the three supervisors’ coaching identities reveal how varied enactments of the UBCF spaces can foster distinct forms of pre-service teacher development. Sylvia’s supportive/stabilizing identity nurtured emotional grounding but was less likely to promote deep cognitive growth. Beth’s guiding/collaborative identity balanced reassurance with cognitive challenge, facilitating emerging metacognition. Ava’s developmental/challenging identity fostered sophisticated reflection and autonomy. These identities were not static but emerged from how each supervisor navigated the relational and developmental demands of their pre-service teachers.

Overall, the findings illustrate how supervisors’ enactments of the UBCF spaces contribute to distinct emotional and reflective developmental trajectories for pre-service teachers. The analysis highlights the importance of coaching identity, the relational flexibility of the UBCF, and the role of adaptive supervisory discourse in supporting teachers’ movement through the reflective–emotional cycle.

## 5. Discussion

The purpose of this study was to understand how university-based supervisors enacted the relational spaces of the UBCF and how these enactments contributed to their approaches to pre-service teachers’ emotional and reflective development. Across the three cases, supervisors constructed distinct coaching identities (supportive/stabilizing, guiding/collaborative, and developmental/challenging) that varied in their intensity of secure, collaborative, and developmental space enactment. The findings demonstrate that supervisory discourse operates simultaneously as an emotional and pedagogical tool, influencing not only how pre-service teachers make sense of their instructional practice but also how they navigate the emotional demands of learning to teach.

### 5.1. Coaching Identities as Relational and Developmental Constructs

The findings illuminate the important role coaching identities play in shaping pre-service teachers’ reflective emotional and knowledge development trajectories. Each supervisor enacted the relational spaces of the UBCF in a patterned and recognizable way that reflected a particular coaching identity adapted to the pre-service teacher’s developmental readiness. These identities emerged organically from the flow of coaching conversations rather than being predetermined stances.

Sylvia’s supportive/stabilizing identity aligned with literature on early-career emotional vulnerability and the importance of psychological safety in novice teacher learning (Hascher et al., 2021; Mansfield et al., 2016). Her heavy reliance on the secure space served an important emotional function: it helped mitigate stress and normalize uncertainty, enabling the pre-service teacher to remain open to feedback during a period of low confidence. Yet this stabilizing function also came with limitations. Because Sylvia relied so strongly on reassurance and emotional buffering, her pre-service teacher engaged only in early-stage reflection characterized by descriptive recounting rather than analytic reasoning. This suggests that overuse of the secure space may inadvertently constrain opportunities for deeper professional learning, a nuance not fully examined in previous UBCF research (Morris & Ogodo, 2024).

Beth’s guiding/collaborative identity represented a transitional pattern of supervision that mirrored the pre-service teacher’s intermediate level of readiness. Her coaching reflected a form of co-constructed reflection, in which analytic reasoning is jointly developed through supportive inquiry and shared problem solving, as supervisors use dialogic prompts and relational scaffolding to guide teachers’ sensemaking (Costa & Garmston, 2015; Haneda et al., 2019). Beth’s balanced integration of secure and collaborative spaces, along with moderate developmental space moves, supported the pre-service teacher’s emerging metacognition without overwhelming her emotionally. This aligns with sociocultural views of learning, in which novices expand their reflective capacity through mediated dialog with a more experienced partner (Schön, 1983).

Ava enacted a developmental/challenging identity that supported high-level reflection and emotional autonomy. Her discourse relied heavily on developmental space moves, characterized by probing questions, analytic prompts, and invitations for professional agency. These patterns reflect coaching approaches aimed at advanced practitioners, where the supervisor operates as a thought partner rather than an emotional anchor (Knight, 2007). Ava’s limited use of the secure space is consistent with research showing that advanced pre-service teachers often benefit more from cognitive challenge than emotional buffering when they have already internalized foundational confidence (Belay & Melesse, 2025). Her coaching stance enabled her pre-service teacher to move into deeper interpretive reasoning and to regulate emotions without external reassurance.

Taken together, these identities demonstrate that supervisors adapt their relational stance in response to pre-service teacher readiness, shaping the emotional and reflective pathways each teacher followed. Viewed through the reflective–emotional cycle, Sylvia’s supportive/stabilizing identity relied heavily on appraisal, enabling the pre-service teacher to name and normalize emotions. Beth’s guiding/collaborative identity balanced appraisal with coping and strategy activation, supporting the pre-service teacher in linking emotions to pedagogical strategies, while Ava’s developmental/challenging identity strongly facilitated reappraisal, prompting the pre-service teacher to reinterpret events and solidify professional agency. This mapping underscores how coaching identities operate as emotional-processing pathways within the reflective–emotional cycle, showing that emotional and cognitive scaffolding co-evolve in relational practice within the UBCF.

### 5.2. Cross-Case Insights: Movement Through UBCF Spaces

The cross-case comparison revealed that distinct coaching identities correspond to adapted enactments of the UBCF spaces, shaping varied pathways through what this study conceptualizes as the reflective–emotional cycle. Intensity patterns, summarized in Table 2, illuminate how supervisors distributed emotional, cognitive, and relational demands within their discourse.

Supervisors differed notably in their dominant space enactment. Sylvia enacted the secure space most intensely, suggesting that emotional containment is a primary need for novice pre-service teachers who are still forming professional confidence. Beth enacted the collaborative space most strongly, reflecting a transitional role in which the pre-service teacher is beginning to co-analyze instructional decisions. Ava relied heavily on the developmental space, supporting autonomy and advanced reflective engagement.

This cross-case pattern illuminates not only differential enactment of the UBCF spaces but also distinct emotional-processing trajectories as described in the AWaRE Model. Sylvia supported appraisal, helping her pre-service teacher surface and articulate emotions. Beth guided the pre-service teacher from appraisal into coping and strategy activation, pairing emotional support with strategic adjustment. Ava’s coaching emphasized reappraisal, pushing the pre-service teacher to reinterpret instructional moments and derive new meaning. These differences illustrate how movement across relational spaces corresponds with progression through the reflective–emotional cycle, reinforcing the idea that coaching is a relationally adaptive and emotionally mediated process.

Importantly, the findings suggest that the UBCF spaces are not linear stages but adaptive relational responses. Supervisors often shifted their coaching stance within a single conversation in response to emotional cues, cognitive readiness, and situational demands, reflecting the dialogic and interactional nature of coaching practice (Costa & Garmston, 2015; Haneda et al., 2019). This finding reinforces prior arguments that coaching is inherently relational and dynamic rather than procedural or sequential (Atkinson et al., 2021). These findings meaningfully extend the UBCF by demonstrating that supervisors do not simply enact discrete feedback strategies but construct multi-layered relational identities that blend emotional and cognitive supports. These identities illuminate how the UBCF spaces operate not only as relational conditions but as mechanisms for supporting teacher knowledge development and emotional resilience.

Moreover, the findings reinforce sociocultural perspectives on teacher knowledge development, which frame learning as relational, discursive, and emotionally mediated (Schön, 1983). The coaching identities documented here show how supervisors scaffold pre-service teachers’ participation in reflective talk, support shifts in professional reasoning, and foster the emotional grounding necessary for complex cognitive work. This supports the argument that emotional and reflective dimensions of teacher learning cannot be separated; they operate interdependently within coaching interactions.

### 5.3. Contributions to Research and Practice

This study offers several contributions to the literature on teacher supervision and coaching. First, it introduces the concept of coaching identities grounded in the UBCF spaces, highlighting the relational and emotional dimensions of supervisory practice. Second, it demonstrates that supervisors’ relational stances shape pre-service teachers’ reflective engagement and emotional resilience in ways not fully captured in existing coaching models. Third, it proposes intensity-based analysis as a method for examining feedback and relational moves in qualitative coaching research, preserving the interpretive depth of discourse analysis while providing a structured comparative framework.

From a theoretical perspective, this study advances scholarship on teacher supervision by specifying interactional and relational mechanisms through which reflective growth and emotional regulation are jointly supported during coaching conversations. By demonstrating how supervisors’ coaching moves enact secure, collaborative, and developmental spaces that facilitate cycles of appraisal, coping, and reappraisal, the study connects affective and cognitive dimensions of teacher learning as interdependent processes. It further reframes coaching identity as emotionally mediated and responsive rather than as a fixed coaching style or set of discrete strategies.

For practice, these findings underscore the importance of preparing supervisors to flexibly navigate relational spaces. Supervisor training should include strategies for recognizing pre-service teacher emotional cues, adapting feedback based on readiness, and sustaining reflective rigor without compromising psychological safety. Programs may also benefit from explicitly teaching supervisors how to construct and shift coaching identities to support pre-service teacher movement through the reflective–emotional cycle. These insights can inform the design of university-based supervision, clinically oriented teacher preparation, and induction coaching.

### 5.4. Limitations

Several limitations warrant consideration. First, the study included only three cases from a single teacher preparation program each during a single coaching session, which limits transferability. Second, while intensity-based coding provides a meaningful comparative structure, it does not capture the full complexity of discourse patterns that might emerge from fine-grained linguistic analysis. Third, as a university-based supervisor, the researcher’s positionality may have shaped interpretations, although reflexive memoing and iterative coding were used to mitigate this influence. Finally, the coaching identities identified here may shift over time or in response to different pre-service teachers, suggesting the need for longitudinal study.

### 5.5. Future Research

Future research should examine coaching identities across larger and more diverse samples, as well as across multiple program contexts. Longitudinal studies could illuminate how coaching identities evolve as supervisors develop expertise or encounter pre-service teachers with varying readiness levels. Additionally, mixed-methods designs, including physiological or emotion-tracking tools, could deepen understanding of how emotional and reflective processes co-occur in coaching interactions. Finally, research examining how pre-service teachers perceive and internalize supervisors’ relational moves would provide important insight into how coaching identities shape learning from the learner’s perspective.

## 6. Conclusions

This study illuminates the relational complexity of university-based coaching by examining how supervisors enact the secure, collaborative, and developmental spaces of the UBCF to shape pre-service teachers’ emotional and reflective development. Across cases, supervisors constructed distinct coaching identities aligned with the developmental needs and emotional readiness of their pre-service teachers. These identities emerged organically through supervisors’ patterned relational moves, illustrating the adaptive and contingent nature of effective coaching.

A key contribution of this study is the demonstration that emotional and reflective processes are intertwined in early teacher learning. Supervisors supported cycles of appraisal, coping and strategy activation, and reappraisal consistent with the AWaRE Model (Hascher et al., 2021) while simultaneously facilitating reflective inquiry that advanced professional reasoning. By linking these emotional processes with the reflective–emotional cycle, the study offers an integrated view of how resilience and reflective depth develop in tandem during supervision.

The findings also extend the UBCF by showing how its relational spaces serve as developmental environments that can be intentionally adjusted to match pre-service teacher needs. Secure grounding supported novices, collaborative meaning-making supported intermediate learners, and developmental challenge promoted autonomy for more advanced practitioners. These insights position the UBCF as a flexible framework capable of guiding differentiated coaching.

Practically, the study highlights the importance of preparing supervisors to navigate emotional attunement and adaptive relational practice. Supervisor preparation should include opportunities to analyze coaching dialogs, reflect on coaching identities, and intentionally shift relational stances. The study additionally contributes a methodological innovation through intensity-based coding, which maintains the depth of qualitative analysis while enabling meaningful cross-case comparison of relational feedback.

Overall, this study advances understanding of the relational foundations of teacher learning by demonstrating how coaching identities support emotional resilience and reflective growth. By aligning supervisors’ relational enactments with the reflective–emotional cycle, the study shows how coaching can guide pre-service teachers through appraisal to coping and strategy activation to reappraisal, fostering both resilience and reflective autonomy. These findings affirm that effective coaching is not simply the delivery of feedback but the creation of relational spaces in which beginning teachers can think bravely, feel supported, and develop the confidence needed to thrive in the profession.

## Figures and Tables

**Table 1:** Main coaching feedback approaches and relational functions.

Feedback Type or Mechanism	Description and Key Features
Supportive Feedback	Emphasizes validation, empathy, and reassurance; builds trust and rapport
Directive Feedback	Provides specific suggestions, corrective modeling, and explicit guidance
Reflective Engagement	Uses metacognitive talk, inquiry questioning, and dialogic feedback to foster reflection
Emotional Regulation	Involves reframing, stress acknowledgment, and coping language to address emotions

Note: Derived from literature (Atkinson et al., 2021; Ennis et al., 2020; Hammond & Moore, 2018; Haneda et al., 2019).

**Table 2:** AWaRE Model components integrated within the UBCF.

AWaRE Model Component	Coaching Space	Function in the Reflective–Emotional Cycle
Appraisal of and Emotions Regarding Event	Secure Space	Emotional Appraisal within a psychologically safe, secure space: Teacher acknowledges emotional responses to classroom stressors in a psychologically safe environment; promotes vulnerability and trust.
Resilience Strategies	Collaborative Space	Coping and Strategy Activation via collaborative, co-constructed planning, and support: Coping actions are co-constructed with the coach, enabling joint problem-solving, rehearsing new strategies, and goal setting.
Appraisal of Emotions Regarding Outcome	Developmental Space	Reappraisal and Wellbeing Restoration through developmental reflection and identity building; The protective factors such as self-efficacy, values, and social-emotional skills are nurtured throughout coaching, especially as the teacher builds self-awareness and professional identity.
Environment with Contextual and Individual Challenges and Resources	The Coaching Relationship Itself	Coaching acts as a buffering environment, a deliberately designed relational context that promotes reflective and adaptive functioning.

**Table 3:** Relative intensities across cases.

Code Category	Sylvia (Novice Pre-Service Teacher)	Beth (Intermediate Pre-Service Teacher)	Ava (Advanced Pre-Service Teacher)
Supportive Feedback	High	Moderate	Low-Moderate
Directive Feedback	Moderate-High	Moderate	Low
Reflective Prompts/ Inquiry	Low-Moderate	Moderate-High	High
Emotional Regulation Indicators	High	Moderate	Moderate
Secure Space Indicators	High	Moderate	Brief but Present
Collaborative Space Indicators	Low-Moderate	High	High
Developmental Space Indicators	Low	Moderate	High

Note: Intensity indicators were applied interpretively and informed by reflexive memos and post-conference analytic conversations with supervisors regarding coaching intent and context.

## Data Availability

Data is stored in university-secured storage. Data may be available upon request.

## Appendix A

**Table A1:** Categories, codes, and descriptions.

Code Category	Example Codes	Description
Supportive Feedback	Validation of effort: “You’re doing fine for where you are.” Normalization of struggle: “That’s completely normal at this stage.” Confidence bolstering: “You handled that better than you think.” Relational reassurance: “I’m proud of how you managed that moment.”	Applied when the supervisor offers emotional encouragement, reassurance, or affirmation aimed at reducing anxiety, increasing confidence, or maintaining psychological safety.
Directive Feedback	Corrective guidance: “Next time, move closer before giving the direction.” Concrete procedural advice: “Try a pair-share if students aren’t responding.” Explicit modeling: “Say it like this: ‘Eyes on me in three…two…’” Focused adjustment: “You need to project your voice a little more here.”	Applied when the supervisor provides explicit, actionable suggestions intended to adjust or improve instructional practice. Tone may be gentle or firm, but content is specific and teacher-facing.
Reflective Prompts/Inquiry	Metacognitive prompting: “What were you noticing when that happened?” Evidence-based questioning: “Do you think that was too many examples?” Alternative generation: “What else could you try next time?” Causal reasoning: “Why do you think they disengaged at that moment?”	Applied when the supervisor invites the pre-service teacher to analyze, reason, infer, or generate alternatives about instructional decisions. Prompts deepen reflection and foster cognitive ownership.
Emotional Regulation Indicators (Appraisal to Coping/Strategy Activation to Reappraisal)	Appraisal: Affect identification: “You seemed overwhelmed. What was happening for you in that moment?” Surfacing emotional cues: “I noticed you paused. Were you feeling unsure there?” Naming emotional experience: “It sounds like you were frustrated when the group didn’t respond.” Coping and Strategy Activation: Immediate grounding: “Take a breath; let’s look at what actually happened.” Emotion-to-action reframing: “When you start feeling rushed, try resetting their attention first.” Stress mitigation strategies: “If participation drops, use a quick pair-share to get them re-engaged.” Reappraisal: Meaning reconstruction: “It felt chaotic, but students were actually tracking with you.” Strengths-focused reinterpretation: “That moment wasn’t a failure; it shows you’re adjusting to their pace.” Emotion-cognition integration: “Looking back, what does this tell you about your developing teaching style?”	Applied when the supervisor helps the pre-service teacher progress through the AWaRE emotional cycle beginning with appraisal (recognizing and naming emotional responses), moving toward coping/strategy activation (regulating emotion and activating next-step strategies), and culminating in reappraisal (reinterpreting the event in a more constructive, agentic way). These indicators reflect how supervisors guide emotional processing as part of reflective pedagogy.
Secure Space Indicators	Invitations for vulnerability: “Tell me how you felt in that moment.” Psychological safety building; “It’s okay that you’re unsure; that’s expected.” Nonjudgmental stance: “Let’s just explore what happened together.” Warm tone and reassurance: consistent affective buffering	Applied when discourse establishes trust, safety, or emotional grounding. High presence in novice-level coaching.
Collaborative Space Indicators	Shared problem solving: “Let’s look at this part together and figure out why that happened.” Co-analysis: “We both noticed the group in the back disengaging—what do you make of that?” Mutual inquiry: “What if we try this strategy next time?” Dialogic sense-making: supervisor and PST build explanations jointly	Applied when the supervisor and pre-service teacher jointly construct understanding, co-interpret data, or co-develop instructional strategies. Tone is egalitarian and inquiry driven.
Developmental Space Indicators	Autonomy-oriented challenge: “What’s your plan for improving this next time?” High-level analytic push: “How does this choice align with your learning goals for the class?” Professional agency prompts: “Is there anything you want to change despite the curriculum constraints?” Identity-focused questioning: “What does this moment tell you about the teacher you’re becoming?”	Applied when the supervisor intentionally pushes for deeper analysis, autonomy, or professional identity formation. Central in advanced PST coaching.

**Table A2:** Intensity-based coding indicators.

Intensity Level	Emotional Regulation Indicators	Reflective Support Indicators
Low Intensity	Emotional cues are implicit or minimally acknowledged; affective language is absent or brief and not taken up as a focus of the interaction. Emotional responses are not reframed or explored.	Reflection is prompted indirectly or superficially (e.g., brief evaluative questions or statements) with limited opportunity for teacher sensemaking.
Moderate Intensity	Emotional responses are explicitly acknowledged and normalized, but emotional regulation is embedded within instructional feedback or problem-solving rather than foregrounded.	Reflective prompts encourage analysis of practice, but sensemaking remains largely scaffolded or guided by the supervisor.
High Intensity	Emotional responses are explicitly named, reframed, or regulated; instructional critique is temporarily suspended to support emotional processing, reassurance, or reappraisal.	Reflection is foregrounded as the primary goal of the interaction; responsibility for analysis, interpretation, and goal-setting is intentionally shifted to the teacher.

Note. Intensity indicators were applied interpretively and used as analytic guides rather than fixed criteria. Judgments were informed by iterative discourse analysis, reflexive memos and post-conference analytic conversations with supervisors regarding coaching intent and contextual factors.
